# CD133 expression is associated with less DNA repair, better response to chemotherapy and survival in ER-positive/HER2-negative breast cancer

**DOI:** 10.21203/rs.3.rs-4148608/v1

**Published:** 2024-03-27

**Authors:** Takumi Sato, Masanori Oshi, Jing Li Huang, Kohei Chida, Arya Mariam Roy, Itaru Endo, Kazuaki Takabe

**Affiliations:** University of Tokyo Hospital; Yokohama City University Graduate School of Medicine; Roswell Park Comprehensive Cancer Center; Roswell Park Comprehensive Cancer Center; Roswell Park Comprehensive Cancer Center; Yokohama City University Graduate School of Medicine; Roswell Park Comprehensive Cancer Center

**Keywords:** CD133, cancer stem cell, cell surface marker, HR positive breast cancer

## Abstract

**Purpose:**

CD133, a cancer stem cells (CSC) marker, has been reported to be associated with treatment resistance and worse survival in triple-negative breast cancer (BC). However, the clinical relevance of CD133 expression in ER-positive/HER2-negative (ER+/HER2−) BC, the most abundant subtype, remains unknown.

**Methods:**

The BC cohorts from the Molecular Taxonomy of Breast Cancer International Consortium (METABRIC, n = 1904) and The Cancer Genome Atlas (TCGA, n = 1065) were used to obtain biological variables and gene expression data.

**Results:**

Epithelial cells were the exclusive source of CD133 gene expression in a bulk BC. CD133-high ER+/HER2− BC was associated with CD24, NOTCH1, DLL1, and ALDH1A1 gene expressions, as well as with WNT/β-Catenin, Hedgehog, and Notchsignaling pathways, all characteristic for CSC. Consistent with a CSC phenotype, CD133-low BC was enriched with gene sets related to cell proliferation, such as G2M Checkpoint, MYC Targets V1, E2F Targets, and Ki67 gene expression. CD133-low BC was also linked with enrichment of genes related to DNA repair, such as BRCA1, E2F1, E2F4, CDK1/2. On the other hand, CD133-high tumors had proinflammatory microenvironment, higher activity of immune cells, and higher expression of genes related to inflammation and immune response. Finally, CD133-high tumors had better pathological complete response after neoadjuvant chemotherapy in GSE25066 cohort and better disease-free survival and overall survival in both TCGA and METABRIC cohorts.

**Conclusion:**

CD133-high ER+/HER2− BC was associated with CSC phenotype such as less cell proliferation and DNA repair, but also with enhanced inflammation, better response to neoadjuvant chemotherapy and better prognosis.

## Introduction

Although cancer stem cells (CSC) comprise only 0.1–1% of the cancer cells within a bulk tumor, they possess properties of self-renewal, initiate tumors from a single cell, and differentiate to resist treatments, thereby being implicated in causing relapses [1–3]. The roles of CSC in breast cancer (BC) have been studied [4–6]; however, the results were sometimes contradictory or incongruent due to heterogeneity in the techniques used to detect CSC populations [7–9]. For example, some studies utilized protein expression, often detected by immunohistochemistry, while others relied on gene expressions of multiple markers.

While many studies used CD44 and CD24 cell surface markers as well as Aldehyde Dehydrogenase (ALDH) to identify CSCs [4, 10–12], CD133 has also been well-characterized as a cell surface marker of CSCs [13]. CD133 was shown to activate WNT/β Catenin pathway in vitro, which is an essential signaling pathway for cell proliferation of CSCs [14]. CD133 also activates the NOTCH and Hedgehog pathways which are other characteristic pathways related to CSCs [15].

Expression of CD133 assessed by immunohistochemistry in 67 patients was shown to correspond to the aggressiveness of triple-negative breast cancer (TNBC) [9]. CD133 was associated with a less response to neoadjuvant chemotherapy (NAC) in 102 BC patients when measured by immunohistochemistry [16]. CD133 mRNA overexpression was linked with a poor prognosis in invasive BC [17]. On the other hand, the clinical significance of CD133 expression in the ER-positive/HER2-negative (ER+/HER2−) BC remains unexplored. Despite the distinct biology of each BC subtype and considering that ER+/HER2− is the most prevalent subtype, there is a gap in our understanding of the specific implications of CD133 in this context.

Our group has been employing an in-silico approach to conduct translational research, investigating the clinical relevance of gene expression. Unlike experiments involving cell lines or animals, we have gained comprehensive and reliable insights by analyzing multiple independent large patient cohorts of transcriptomes associated with clinical parameters [18–21]. In this study, we hypothesize that high CD133 expression would be associated with the prognosis of ER+/HER2− BC. To better elucidate the association between the prognosis of ER+/HER2− BC and the CSC surface marker CD133, we utilized two distinct cohorts: The Cancer Genome Atlas (TCGA) cohort, which includes 1065 breast cancer patients, and the Molecular Taxonomy of Breast Cancer International Consortium (METABRIC) cohort, consisting of 1904 breast cancer patients.

## Methods

### Acquisition of BC patients’ data

The gene expression data associated with clinical parameters was obtained from 1065 patients of The Cancer Genome Atlas (TCGA) BC cohort and 1904 patients of the Molecular Taxonomy of Breast Cancer International Consortium (METABRIC) cohort [22]. In accordance with previous studies, cBioportal was utilized to identify the cohorts which included mutation status (https://www.cbioportal.org) [13, 18, 23–35]. According to the staging guidelines of the American Joint Committee on Cancer, BC staging was conducted. Other BC cohorts analyzed in this study include cohorts of patients who received neoadjuvant chemotherapy (NAC); GSE25066 [36], GSE20194 [37], GSE32646 [38], and single cell sequence cohorts; SCP1039 (https://singlecell.broadinstitute.org/single_cell/study/SCP1039/a-single-cell-and-spatially-resolved-atlas-of-human-breast-cancers) and SCP1106 (https://singlecell.broadinstitute.org/single_cell/study/SCP1106/stromal-cell-diversity-associated-with-immune-evasion-in-human-triple-negative-breast-cancer). We obtained a waiver of the Roswell Park Institutional Review Board for approval, given the de-identified nature of the data points.

### Gene set enrichment analysis (GSEA)

GSEA investigates the extent to which the expression of genes related to a certain pathway differs between groups. In this study, the cohort was categorized into ‘high’ and ‘low’ expression groups based on the median value as the cutoff. Fifty Hallmarks of Cancer gene sets in the Molecular Signatures Database (MSigDB) [39] were studied, as previously demonstrated by the Broad Institute (http://www.gsea-msigdb.org/gsea/index.jsp) [40]. The Normalized Enrichment Score (NES) was employed to assess the strength of the correlation. The False Discovery Rate (FDR) was utilized for statistical analysis, considering a cutoff for significance as an FDR value of less than 0.25. This choice aligns with the recommendation by the Broad Institute to adjust for gene set size, considering the multiple gene sets analyzed in our study.

### Cell composition analysis

Thorsson et al. computed and reported cell proliferation score, homologous recombination defects score, mutation rate score, neoantigens score, and immune activity score [41]. We utilized xCell, the web-based computational algorithm developed at the University of California San Francisco (https://www.xcell.ucsf.edu), to analyze the association between the transcriptomic data and the enrichment of immune cells in different BC groups, as previously described [24–25, 27–31, 42–44]. Transcriptomic data of 64 cell types in the tumor microenvironment (TME) can be analyzed by the xCell algorithm. In this study, we analyzed transcriptomic data of immune cells such as T helper cells [45], regulatory T cells, M1 and M2 macrophages, CD8 + cells, CD4 memory cells [23], dendritic cells [24], and B cells.

### Statistical Analysis

Data downloading, analysis, organization, and visualization were performed using R software (version 4.0.1. http://www.r-project.org/). Histograms were created to describe differences between “high” and “low” CD133 tumors. Two-sided test was employed to calculate p-values, and the cutoff of less than 0.05 was regarded as statistically significant. Median and interquartile level values were displayed using Tukey-type boxplots. Survival analyses were performed using Kaplan-Meier plots with log-rank tests.

## Results

### CD133 gene was predominantly expressed in epithelial cells in the BC tumor microenvironment (TME) of single-cell sequence cohorts

**Figure 1:**
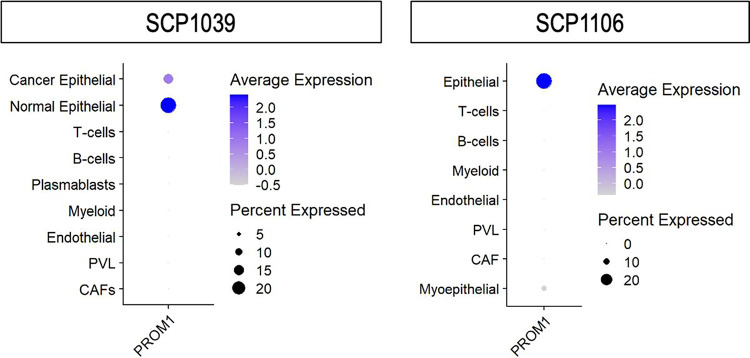
CD133 gene expression levels and percentages of cells expressed in single-cell sequence cohorts, SCP1039 and SCP1106. CD133 expression pattern in various cell traits (cancer/normal epithelial cells, plasmablasts, T-cells, B-cells, endothelial cells, myeloid cells, PVL, and CAFs) in SCP1039 and SCP1106 cohorts. PVL, perivascular-like subpopulations: CAFs, Cancer-Associated Fibroblasts.

Given that CD133 is a well-characterized cell surface marker of CSCs [13], we first investigated which type of cells in the TME express CD133. This analysis was conducted using two independent single-cell sequence cohorts of BC patients, SCP1039 and SCP1106. We found that epithelial cells, from which BC cells arise, predominantly express CD133 in the TME (p < 0.001, Fig. 1). Since epithelial cells in bulk tumors are almost exclusively cancer cells, the observation of high CD133 expression in “normal” epithelial cells in SCP1039 likely represents less proliferative cancer cells.

### CD133 expression was associated with CSC markers and stemness-related signaling pathways

**Figure 2:**
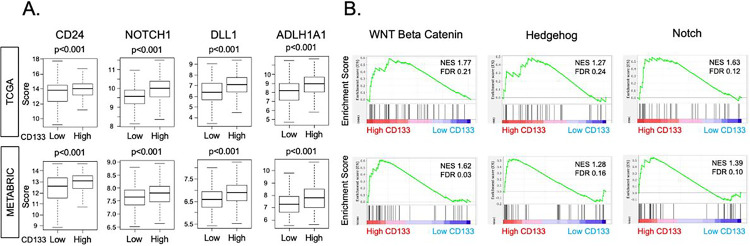
The relationship between CD133 expression and CSC surface marker and stemness-related signaling pathways in TCGA and METABRIC cohorts. **(A)** Gene expression levels of CSC markers (CD24, NOTCH1, DLL1, ALDH1A1) by high or low CD133 expression in TCGA and METABRIC cohorts are given in boxplots. The cohort was divided into “high” and “low” expression groups by the median value. Two-sided test was employed to calculate p-values, and the cutoff of less than 0.05 was regarded as statistically significant. The vertical line in the box shows the median, and top and bottom show the 25^th^ and the 75^th^ percentiles, respectively. **(B)** Gene set enrichment analysis (GSEA) of stemness-related signaling pathways; WNT/beta-catenin, Hedgehog, NOTCH. The classical GSEA method was used to compute NES and FDR values, with a cutoff of FDR < 0.25 considered statistically significant. CSC, cancer stem cells: NES, normalized enrichment score: FDR, false discovery rate.

Several CSC markers and their characteristic signaling pathways have been reported [6], and CD133 was reported to be one of them in colon [13, 46] and ovarian cancer [47]. Consequently, we sought to explore whether CD133 expression is associated with other CSC markers and stemness-related signaling pathways in ER-positive/HER2-negative (ER+/HER2−) BC. We found that CD133-high BC was significantly associated with higher expression levels of CSC marker genes: CD24, NOTCH1, DLL1, and ALDH1A1, consistently in both TCGA and METABRIC cohorts (all p < 0.001, Fig. 2A). Furthermore, we found that CD133-high BC significantly enriched signaling pathways that are activated in CSCs: WNT/β catenin signaling (normalized enrichment score (NES) = 1.77, FDR = 0.21), Hedgehog signaling (NES = 1.27, FDR = 0.24), and NOTCH signaling (NES = 1.63, FDR = 0.12) in TCGA, which was validated in the METABRIC cohort: WNT/β-catenin signaling (NES = 1.62, FDR = 0.03), Hedgehog signaling (NES = 1.28, FDR = 0.16), and NOTCH signaling (NES = 1.39, FDR = 0.10) (Fig. 2B). These results suggest that CD133 is associated with other CSC markers and their signaling pathways in ER+/HER2− negative BC.

### CD133 expression was negatively associated with cancer cell proliferation

**Figure 3:**
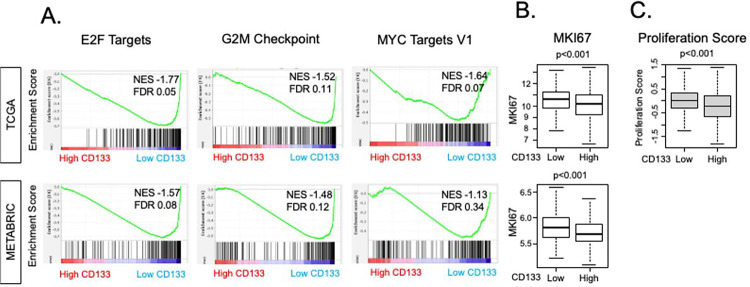
Association of CD133 expression with cell proliferation-related pathways, gene expression, and a score. **(A)** Gene set enrichment analysis (GSEA) of cell proliferation-related hallmark gene sets; E2F Targets, G2M Checkpoint, and MYC Targets version 1. The classical GSEA method was employed to calculate NES and FDR values, and the cutoff of FDR < 0.25 was regarded as statistically significant. **(B)** Boxplots showing the expression level of MKI67 of low and high CD133 expression groups in the TCGA and METABRIC cohorts. The cohort was divided into “high” and “low” expression groups by the median value. A two-sided test was employed to calculate p-values, and the cutoff of less than 0.05 was regarded as statistically significant. **(C)** Boxplot of the proliferation score that Thorson et al. pre-calculated.

Since CSCs are known to be less proliferative compared to differentiated cancer cells [3, 48–51], we were interested in whether the same trend holds true for CD133 expression in ER+/HER2− BC. Indeed, we found that Hallmark gene sets related to cell proliferation: E2F Targets (NES = −1.77, FDR = 0.05), G2M Checkpoint (NES = −1.52, FDR = 0.11), and MYC Targets V1 (NES = −1.64, FDR = 0.07) were all significantly enriched in CD133-low BC in the TCGA cohort (Fig. 3A). Enrichment of E2F Targets (NES = −1.57, FDR = 0.08) and G2M Checkpoint (NES = −1.48, FDR 0.11), but not MYC Targets V1, was validated in the METABRIC cohort. Furthermore, CD133-low tumors were associated with higher Ki67 gene (MKI67) expression compared to CD133-high tumors consistently in both cohorts (both p < 0.001, Fig. 3B). In concurrence with this, the pre-calculated Proliferation Score by Thorsson et al. [41] demonstrated an inverse relationship with CD133 expression in the TCGA cohort (p < 0.001, Fig. 3C). These findings compellingly suggest that elevated CD133 expression is linked to lower cell proliferation in ER+/HER2− BC.

### CD133 expression was associated with less DNA repair activity and less mutation rate

**Figure 4:**
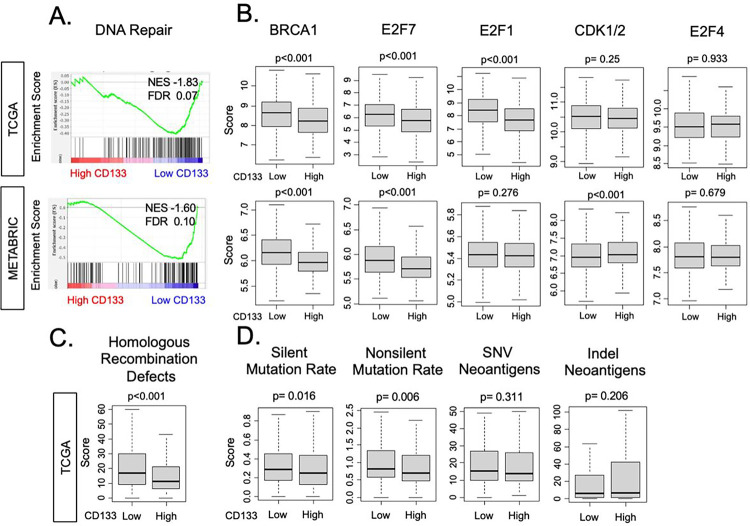
Association of CD133 expression with DNA-repair pathway, related gene expression, and a score. **(A**) Gene set enrichment analysis (GSEA) of DNA-repair pathway. The classical GSEA method was employed to calculate NES and FDR values, and the cutoff of FDR < 0.25 was regarded as statistically significant. **(B)** Boxplots illustrating the gene expression levels of DNA repair-related genes (BRCA1, E2F1, E2F4, E2F7, and CDK 1/2) in the low and high CD133 expression groups within the TCGA and METABRIC cohorts. Two-sided test was employed to calculate p-values, and the cutoff of less than 0.05 was regarded as statistically significant. **(C)** The boxplot of Homologous Recombination Defects scores that Thorson et al. pre-calculated, by low and high CD133 expression in the TCGA cohort. The cohort was divided into “high” and “low” expression groups by the median value. **(D)** Boxplots illustrating the levels of SNV neoantigens, indel neoantigens, silent mutation rate, and non-silent mutation rate in the low and high CD133 expression groups within the TCGA cohort.

We have previously demonstrated that DNA repair pathway enhancement is linked with cell proliferation [18, 45, 52]. Given that CD133 expression was negatively associated with cell proliferation in ER+/HER2− BC, it was of interest to explore whether CD133 gene expression was associated with the DNA repair pathway and its related gene expressions. We found that DNA repair gene set was significantly enriched in CD133-low tumors in both TCGA (NES = −1.83, FDR = 0.07) and METABRIC cohorts (NES = −1.60, FDR = 0.10, Fig. 4A), aligning with the relationship between CD133 expression and cell proliferation. Expressions of genes related to DNA repair such as BRCA1 and E2F7 were significantly higher in CD133-low tumors in both TCGA and METABRIC cohorts (all p < 0.001, Fig. 4B), whereas E2F1 and CDK1/2 expressions were higher in only one cohort (TCGA and METABRIC, respectively, both p < 0.001), not validated by other. Although E2F4 is also known as a gene related to DNA repair [53], there was no relationship with CD133 expression in our study. Homologous recombination deficiency (HRD) scores were negatively correlated with CD133 expression in the TCGA cohort (Fig. 4C), and silent and non-silent mutation rate was slightly enriched in CD133-low tumors (Fig. 4D). On the other hand, there were no differences in single nucleotide mutations (SNV) neoantigens and indel neoantigens by CD133 expression in the TCGA cohort. These results overall suggest CD133-high ER+/HER2− BC is associated with less DNA repair activity.

### CD133-high tumors were associated with inflammation and immune response-related pathways

**Figure 5:**
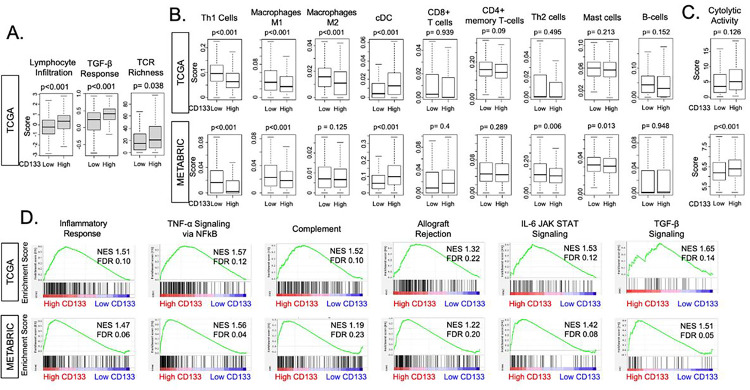
Association of CD133 expression with inflammation and immune response-related pathways. **(A)** The boxplots of scores Thorson et al. pre-calculated regarding pro-inflammatory microenvironment; Lymphocyte infiltrating signature, TGF-β response, and TCR richness. The TCGA cohort was divided into low and high CD133 expression groups by median cut-off. Two-sided test was employed to calculate p-values, and the cutoff of less than 0.05 was regarded as statistically significant. **(B)** Boxplots showing the levels of infiltration by several types of immune cells; Th1 cells, M1 and M2 macrophages, cDC in the TCGA and METABRIC cohorts. **(C)** The boxplots showing cytolytic activity, which summarizes the overall killing ability of immune cells. **(D)** Gene set enrichment analysis (GSEA) of inflammation and immune response-related pathways; inflammatory response, TNFα signaling via NFκB, complement, allograft rejection, IL-6 JAK STAT signaling pathway, and TGFβ signaling pathway. The classical GSEA method was employed to calculate NES and FDR values, and the cutoff of FDR < 0.25 was regarded as statistically significant. TCR, T cell Receptor: cDC, classical dendritic cells.

Upon discerning diminished DNA repair in CD133-high tumors, our focus shifted towards investigating a potential association between CD133 expression and cancer immunity. This inquiry is motivated by our previous findings, which demonstrated a correlation between reduced DNA repair, heightened immunogenicity, and enhanced cancer immunity [45]. Utilizing the scores pre-calculated by Thorsson et al. [41], we found that CD133-high ER+/HER2− BC was associated with greater lymphocyte infiltration, TGF-β response (both p < 0.001), and T cell Receptor (TCR) richness (p = 0.038), suggesting a proinflammatory microenvironment in the TCGA cohort (Fig. 5A). We observed difference in the infiltration of several types of immune cells between CD133-high and low BC, such as Th1 cells, M1 and M2 macrophages which infiltrated less, and classical dendritic cells (cDC) which infiltrated more in CD133-high BC, while many types of immune cells, such as CD8 + T cells, CD4 + memory T cells, Th2 cells, Mast cells, and B-cells, presented no significant difference in both cohorts (Fig. 5B). Cytolytic activity, which encapsulates the collective killing prowess of immune cells, exhibited a positive correlation with CD133 expression in the METABRIC cohort (p < 0.001). The same trend held true in the TCGA cohort, although insignificantly (p = 0.126, Fig. 5C). We further found that many Hallmark gene sets related to inflammation and immune response were enriched in CD133-high tumors in the TCGA cohort: inflammatory response (NES = 1.51, FDR = 0.10), TNF-α signaling via NFκB (NES = 1.57, FDR = 0.12), complement (NES = 1.52, FDR = 0.10), allograft rejection (NES = 1.32, FDR = 0.22), IL6 JAK STAT3 signaling (NES = 1.53, FDR = 0.12) and TGF-β signaling (NES = 1.65, FDR = 0.14), all of which were validated in the METABRIC cohort: inflammatory response (NES = 1.47, FDR = 0.06), TNF-α signaling via NFκB (NES = 1.56, FDR = 0.04), complement (NES = 1.19, FDR = 0.23), allograft rejection (NES = 1.22, FDR = 0.20), IL6 JAK STAT3 signaling (NES = 1.42, FDR = 0.08) and TGF-β signaling (NES = 1.51, FDR = 0.05, Fig. 5D). These results suggest that CD133 expression is associated with inflammation and immune response in TME of ER+/HER2− BC.

### CD133-high tumors were associated with better response to neoadjuvant chemotherapy (NAC) and survival

**Figure 6:**
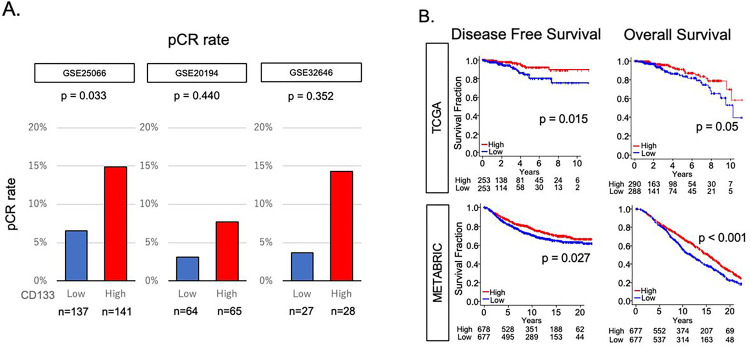
Association of CD133 expression with response to chemotherapy and survival. **(A)** Bar graphs of pCR rate by low and high CD133 expressing patients in GSE25066, GSE20194, and GSE32646 cohorts. The cohorts were divided into “high” and “low” expression groups by the median value. **(B)** Kaplan-Meier curves demonstrating DFS and OS in TCGA and METABRIC cohorts. The blue lines depict the low CD133 expression group, while the red lines represent the high CD133 expression group. Log-rank tests were employed for calculating p-values. pCR, pathological complete response: DFS, disease free survival: OS, overall survival.

Given that CD133 expression was associated with less DNA repair but enhanced inflammation and immune response, we hypothesized that CD133-high ER+/HER2− BC may be vulnerable to cellular insult and respond better to chemotherapy. We utilized GSE25066, GSE20194, and GSE32646 cohorts, which included ER+/HER2− BC patients who underwent NAC: taxane and anthracycline in GSE25066, paclitaxel, 5-fluorouracil, cyclophosphamide, and doxorubicin in GSE20194, and paclitaxel followed by 5-fluorouracil, epirubicin, cyclophosphamide (P-FEC) combination in GSE32646. CD133-high tumors responded significantly better than CD133-low tumors with pathological complete response (pCR) rate of 6.6% vs 14.9% after NAC in the GSE25066 cohort (n = 278, p = 0.03), the largest cohort of the three. Although statistical significance was not achieved, the trend that CD133-high tumors achieved a higher pCR rate was consistent in GSE20194 with a pCR rate of 3.1% vs 7.7% and GSE32646 cohorts with a pCR rate of 3.7% vs 14.3% (n = 129 and 55, respectively, Fig. 6A). CD133-high tumors were associated with better disease-free survival (DFS, p = 0.015) and overall survival (OS, p = 0.05) in the TCGA cohort, and both DFS (p = 0.027) and OS (p < 0.001) were validated by the METABRIC cohort (Fig. 6B).

## Discussion

In summarizing our study, CD133 was exclusively expressed in cancer cells compared to stromal and immune cells and was associated with other CSC markers (CD24, NOTCH1, DLL1, and ALDH1A1), as well as enriched WNT/β-Catenin, Hedgehog, and NOTCH signaling, validating CD133 as a CSC marker. We found that the expression of the cancer stem cell marker CD133 is associated with reduced cell proliferation and DNA repair, yet heightened inflammation, and is linked to a more favorable outcomes after NAC and improved survival among ER-positive/HER2-negative BC patients.

Based on the fact that the CSCs are less proliferative than other types of cells in the tumor, we expected the expression of the CSC marker CD133 to be related to less cell proliferation. However, Joseph et al. reported that CD133 is associated with greater cell proliferation, less response to NAC, and worse prognosis in invasive BC [17]. Our data was consistent with our expectation and contradicted Joseph et al.’s report, which analyzed invasive BC as a whole, as opposed to our study that specifically investigated the ER+/HER2− subtype based on the understanding that biology and characteristics are significantly different by subtypes. It may be worth noting that CD133 protein expression evaluated by flow cytometry did not correlate with its mRNA expression level [54].

Our team, alongside other investigators, has reported an association between DNA repair enhancement and cell proliferation [18, 52]. The same trend has been shown by Oshi et al. in hepatocellular carcinoma [45], who found that enhanced DNA repair was associated with a worse prognosis and more cell proliferation but not with the fraction of immune cell infiltration nor immune response. Consistently, high expressions of RAD51 [18] or BRCA2 [52], both of which play a critical part in DNA repair, were associated with increased cell proliferation and aggressive biology in BC. Given that CD133-high BC was associated with less cell proliferation, its association with less DNA repair may explain its mechanism. On the other hand, Cheah et al. reported that CD133-marked putative CSCs correlated with proficient mismatch repair [55], thus multiple mechanisms may be involved in the relationship between CD133 expression and DNA repair.

We also found that inflammation and immune response were enriched in CD133-high TME. The number of many types of infiltrating cells in TME were not significantly different between high and low CD133 tumors and, interestingly, some types of cells were negatively correlated with CD133 expression. However, cytolytic activity, which represents the overall activity of the immune cells and thus cancer immunity, was significantly and positively correlated with high CD133 expression. It remains unclear precisely how and, after all, whether low DNA repair leads to high inflammation in the TME. Several previous studies reported that in several cell lines and cancer types, low DNA repair led to a higher neoantigen load, therefore high immunogenicity, and, as a result, more lymphocytes infiltration and richer inflammation [38, 56]. Nevertheless, while we observed slightly higher silent and non-silent mutation rates in CD133-low tumors, no discernible difference was noted in SNV neoantigens and indel neoantigens based on CD133 expression. This observation diminishes the persuasiveness of that explanation in our study. However, there are still several possible mechanisms that impaired DNA repair results in richer inflammation in TME, although not in higher loads of neoantigens. One is through the accumulation of DNA damage and subsequent activation of several signaling pathways such as the ATM/ATR pathway and the DNA/PK pathway, which can lead to the activation of NFκB and other pro-inflammatory transcription factors that induce the production of pro-inflammatory cytokines, chemokines, and growth factors by cancer cells and surrounding immune cells [57]. This hypothesis is further supported by the fact that TNFα signaling via NFκB is enriched in CD133-high tumors in our study (Fig. 5C), which is also known to enrich inflammation [58]. Another possible explanation is that impaired DNA repair results in the accumulation of damaged or misfolded proteins in the endoplasmic reticulum of cancer cells, leading to endoplasmic reticulum stress and activation of the unfolded protein response (UPR). The UPR can also activate pro-inflammatory pathways, leading to the production of pro-inflammatory cytokines and chemokines [59–60], although several previous studies suggest that the chronic activation of UPR is considered a mechanism of tumor progression [61–63], going against better DFS and OS observed in our study, which may be due to the difference in cohorts.

Finally, and most importantly, we found that CD133-high BC carried a better survival outcome. We cannot help but speculate that while CD133-high tumors have a poor prognosis, as previous studies suggest accordingly with the cancer stem cell concept that involves self-renewal, differentiation, and the initiation of tumorigenesis, CD133-low tumors may carry even worse prognosis due to their ability to repair DNA, more cell proliferation, decreased immunogenicity, hence less response to NAC and worse survival outcome. The correlation between inflammation and pCR in invasive BC has been proposed by Hatzis et al. [36]. Furthermore, less cell proliferation in CD133-high BC may explain better prognosis, going along with some prior findings that showed an association between more expression of genes related to proliferation such as G2M [26–27], E2F [23, 25], and MYC [64] and worse prognosis in ER+/HER2− BC. In summary, the association of elevated CD133 expression in breast cancer cells with diminished DNA repair, improved response to NAC, and enhanced survival underscores CD133’s potential role as a marker for predicting the treatment response in ER+/HER2− subtype BC.

Our method is subject to certain limitations inherent in the essentially retrospective nature of this study. Firstly, the utilization of patient sample data from a public domain means the analysis relies on information that had previously been cataloged, resulting in limited granularity. Secondly, the origin of the sample within the bulk tumor may vary among patients, even though the spatial relationship of CSCs in the bulk tumor may be of importance. It has been indicated that CSCs at the periphery of the bulk tumor may not have been sampled [65], although CD133 is known to be particularly upregulated in low O2 tissues [66]. These biases may have resulted in an underrepresentation of the full array and functionality of the CSCs.

## Conclusion

CD133, a cancer stem cell (CSC) marker, was associated with less cell proliferation and less DNA repair, but with enhanced inflammation and better response to NAC and enhanced survival in ER+/HER2− BC.

## Data Availability

Publicly available datasets were analyzed in this study. TCGA data can be found here: [https://www.cbioportal.org. /Breast Invasive Carcinoma (TCGA, PanCancer Atlas)]. METABRIC data can be found here: [https://www.cbioportal.org. / Breast Cancer (METABRIC, Nature 2012 & Nat Commun 2016)]. Data sets from each of the GEO databases can be downloaded from the following sites and access numbers: [https://www.ncbi.nlm.nih.gov/geo./GSE96058/GSE2034/GSE124647/GSE159956/GSE12276/GSE110590/GSE20194/GSE25066/GSE163882/ GSE20271].

## Abbreviations

CSC: cancer stem cell
GSEA: Gene set enrichment analysis
TCGA: The Cancer Genome Atlas
METABRIC: Molecular Taxonomy of Breast Cancer International Consortium
OS: overall survival
DFS: disease-free survival
